# Bacteremia due to a rare opportunistic pathogen, *Kluyvera intermedia*, with atypical antimicrobial susceptibility in the absence of β-lactamases

**DOI:** 10.1128/asmcr.00047-25

**Published:** 2025-06-12

**Authors:** Tevan Luong, Anatasia Weiland, Lanny Hsieh, Ayesha Khan

**Affiliations:** 1 School of Medicine, University of California, Irvine 12219, Irvine, California, USA; 2 Division of Infectious Diseases, Department of Medicine, School of Medicine, University of California, Irvine 12219, Irvine, California, USA; 3 Division of Hospital Medicine and Palliative Medicine, School of Medicine, University of California, Irvine 12219, Irvine, California, USA; 4 Division of Clinical Microbiology, Department of Pathology and Laboratory Medicine, School of Medicine, University of California, Irvine 12219, Irvine, California, USA; Vanderbilt University Medical Center, Nashville, Tennessee, USA

**Keywords:** antimicrobial resistance, gram-negative resistance, esbl, *Kluyvera intermedia*, bacteremia, CTX-M, septic shock, cholecystitis, Kluyvera

## Abstract

**Background:**

*Kluyvera* are gastrointestinal commensals and emerging opportunistic pathogens. *Kluyvera* are considered intrinsically resistant to ampicillin and early generation cephalosporins due to chromosomal β-lactamase encoding *bla*_KLU_ genes, which are the proposed ancestors of modern CTX-M enzymes.

**Case Summary:**

Here, we present a case of bacteremia and septic shock in a 61-year-old patient with uncontrolled type 2 diabetes caused by the rare species, *Kluyvera intermedia*. It was misidentified as “*Enterobacter* (non-*cloacae* complex)” by the ePlex blood culture molecular identification panel. The *K. intermedia* displayed atypical susceptibility to all β-lactams that diverge from previous clinical case reports on *Kluyvera*. The patient was successfully treated with piperacillin-tazobactam and surgical source control. Only one clinical case of *K. intermedia* infection has been reported before. We identified three other cases at our institution from 2023 and 2024, including two severe infections. Analysis of 162 publicly available *Kluyvera* genomes on NCBI revealed that chromosomal *bla*_KLU_ genes are not uniformly present in all *Kluyvera* species. Strikingly, chromosomal *bla*_KLU_ genes are present in 0% of *K. intermedia* genomes; 100% of *Kluyvera ascorbata, Kluyvera cryocrescens,* and *Kluyvera sichuanensis* genomes; and 50% of other *Kluyvera* species genomes. There is also extensive diversity in the prevalence of plasmid-mediated extended-spectrum β-lactamases and carbapenemases across *Kluyvera* species.

**Conclusion:**

This case highlights clinically relevant, species-specific genomic variation in *Kluyvera* that impacts antimicrobial susceptibility and treatment. *K. intermedia* has a distinct genomic landscape of antimicrobial resistance than other *Kluyvera* species, suggesting that the origin of CTX-M enzymes may still be unknown. This case underscores the importance of utilizing culture-based identification and phenotypic susceptibility testing to guide the treatment of *Kluyvera* infections.

## INTRODUCTION

*Kluyvera* are poorly characterized commensals in the gastrointestinal tract and rare opportunistic pathogens infrequently associated with human infections including bacteremia, urinary tract infections, cholangitis, and wound infections (1–3). These gram-negative bacilli in the *Enterobacterales* family were historically regarded as benign saprophytes found in soil, food, water, sewage, or animals. In 1981, *Kluyvera* were taxonomically deemed as a distinct genus and have since been increasingly reported as a cause of infections, particularly in immunocompromised patients (3). *Kluyvera ascorbata* is the most common species associated with human infections.

*Kluyvera* species are known to harbor chromosomal, β-lactamase encoding, *bla_KLU_* genes that are considered the ancestral progenitors of modern CTX-M enzymes. CTX-M enzymes are the most globally prevalent extended-spectrum β-lactamases (ESBLs) and are recognized as major public health threats (2–4). Infections caused by ESBL-producing organisms are on the rise (5). *Kluyvera* are considered intrinsically resistant to ampicillin, first- and second-generation cephalosporins, but generally susceptible to third- and fourth-generation cephalosporins, due to the chromosomal *bla_KLU_* encoded β-lactamase. Blood culture molecular identification panels have been reported to falsely detect *bla*_CTX-M-15_ in *Kluyvera*, risking carbapenem misuse and overuse in the absence of a true ESBL (6). *Kluyvera* has also been reported to acquire plasmid-borne genes encoding for ESBLs and carbapenemases like other Enterobacterales (7–12). Advances in whole-genome sequencing have unveiled extensive diversity within bla_KLU/CTX-M_ genes and led to taxonomic rearrangements in the *Kluyvera* genus. This genomic diversity is hypothesized to complicate organism identification in clinical laboratories, potentially underestimating the pathogenic potential of *Kluyvera* (2).

Here, we present a case of bacteremia and septic shock secondary to acute cholecystitis, caused by a rarer species, *Kluyvera intermedia*, which was susceptible to all β-lactams in the absence of β-lactamase production. To our knowledge, this is the second case report of *K. intermedia* infection to date, and the first pan-susceptible strain, underscoring the importance of understanding species-level variations in antimicrobial resistance mechanisms for optimal treatment of invasive infections caused by rare organisms (13).

## CASE PRESENTATION

A 61-year-old man with uncontrolled type 2 diabetes (hemoglobin A1c 13.4%; diabetes range: >6.5%) presented to the emergency department (ED) with a 1-day history of abdominal pain, bloating, nausea, vomiting, and a fever (103°F). On arrival, he was hypotensive (blood pressure of 90/60 mmHg), tachycardic (heart rate of 105 beats/min), but afebrile with normal respiratory rate and oxygen saturation. Physical examination revealed mild distress due to pain, right upper quadrant abdominal tenderness, and a positive Murphy sign. He was empirically started on intravenous (IV) metronidazole (500 mg every 8 h) and IV ceftriaxone (2 g every 24 h). Laboratory evaluation showed leukocytosis with a white blood cell count of 33,000 cells/µL (reference range: 4,000–10,500 cells/µL), hyperglycemia (serum glucose of 529 mg/dL, reference range: 85–125 mg/dL), acute kidney injury (creatinine of 3.4 mg/dL, reference range: 0.7–1.3 mg/dL), lactic acidosis (lactate of 4.8 mmol/L, reference range: 0.5–2 mmol/L), elevated liver enzymes with alkaline phosphatase at 421 U/L (reference range: 34–104 U/L), alanine transferase at 700 U/L (reference range: 7–52 U/L), aspartate transaminase at 726 U/L (reference range: 13–39 U/L), and a normal total bilirubin of 1.4 mg/dL (reference range: 0–1.4 mg/dL). A non-contrasted abdominal computed tomography scan was concerning for acute cholecystitis. The patient was subsequently admitted to the intensive care unit. Empiric therapy was broadened to IV piperacillin-tazobactam (3.375 g every 8 h) and IV vancomycin (dosed to target area under the curve [AUC]/MIC of 400–600 mg*h/L) pending culture results.

Two sets of blood cultures collected in the ED were positive within 16 h of incubation with gram-negative bacilli seen on the gram stain. The ePlex BCID-GN PCR assay (Roche, Indianapolis, IN) was performed and positive for *“Enterobacter* (non-*cloacae* complex)” with no resistance genes detected. The ePlex BCID-GN panel can detect genes encoding for the following β-lactamases: CTX-M-15 (ESBL), KPC (class A serine carbapenemase), OXA-48 (class D serine carbapenemase), and class B metallo-β-lactamases (NDM, VIM, and IMP). Based on these results, piperacillin-tazobactam was continued, and vancomycin was discontinued.

**Fig 1:**
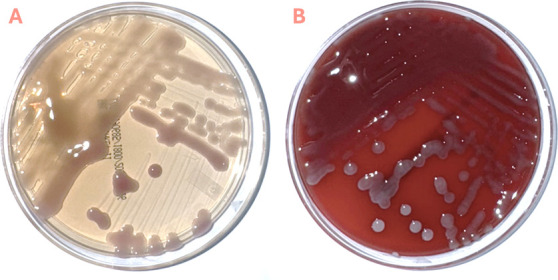
Images of agar plates growing the *K. intermedia* isolate cultured from this patient’s blood. (A) MacConkey agar plate showing light pink, mucoid colonies indicative of slow lactose fermentation. (B) Sheep blood agar plate with gray, mucoid colonies.

**Fig 2:**
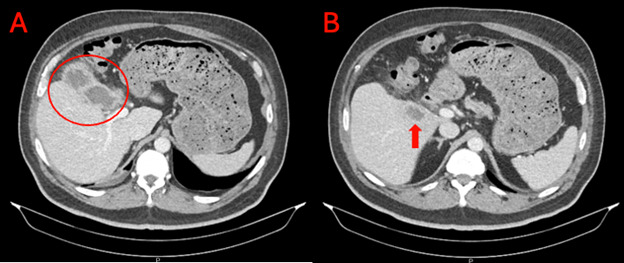
Representative image from a CT scan. (A) A thickened, irregular gallbladder wall with pericholecystic fat stranding representing acute gangrenous cholecystitis. (B) A 2.7-cm rim-enhancing liver hypodensity suggestive of an abscess.

The following day, large, gray-white, mucoid colonies were grown on sheep blood agar with faint lactose fermentation on MacConkey agar (Fig. 1). The organism was identified as *K. intermedia* by matrix-assisted laser desorption ionization-time of flight mass spectrometry (MALDI-TOF MS, Vitek MS, bioMérieux), with a score of 2.28. Phenotypic antimicrobial susceptibility testing was performed on the Vitek2 (bioMérieux). The isolate was susceptible to all antimicrobials, including ampicillin, cefoxitin, and ceftriaxone (Table 1). Repeat blood cultures on hospital day 4 were negative. After a 10-day course of IV piperacillin-tazobactam, the patient was transferred to the medical floor. A follow-up contrast-enhanced abdominal CT scan demonstrated findings suggestive of gangrenous cholecystitis (Fig. 2). The patient underwent a successful robot-assisted laparoscopic cholecystectomy with intraoperative findings of a gangrenous gallbladder with a small volume contained perforation. He was discharged on postoperative day 3 with oral levofloxacin (750 mg daily) and oral metronidazole (500 mg every 8 h) for a 7-day course from the day of surgery.

**TABLE 1:** Antibiotic susceptibility profiles of *K. intermedia* isolated from hospitalized patients hospitalized in a tertiary care center in Southern California between 2014 and 2024^*a*^

Antimicrobial	Isolate 1	Isolate 2	Isolate 3	Isolate 4^*b*^
Ampicillin	≥32 (R)	≥32 (R)	≥32 (R)	4 (S)
Ampicillin-sulbactam	8 (S)	≤2 (S)	≤2 (S)	≤2 (S)
Piperacillin-tazobactam	≤4 (S)	≤4 (S)	≤4 (S)	≤4 (S)
Cefazolin	20 mm (I)^*c*^	13 mm (R)^*c*^	9 mm (R)^*c*^	25 mm (S)^*c*^
Cefotaxime	NT	NT	NT	NT
Cefuroxime	NT	4 (S)	NT	≤1 (S)
Cefepime	≤0.12 (S)	≤0.12 (S)	≤0.12 (S)	≤0.12 (S)
Cefoxitin	≥32 (R)	≥32 (R)	≥32 (R)	4 (S)
Ceftazidime	NT	NT	NT	NT
Ceftriaxone	30 mm (S)	28 mm (S)	30 mm (S)	26 mm (S)
Ciprofloxacin	≤0.25 (S)	≤0.25 (S)	≤0.25 (S)	≤0.25 (S)
Levofloxacin	≤0.12 (S)	≤0.12 (S)	≤0.12 (S)	≤0.12 (S)
Nitrofurantoin^*d*^	NT	16 (S)	NT	32 (S)
Ertapenem	≤0.12 (S)	≤0.12 (S)	≤0.12 (S)	≤0.12 (S)
Imipenem-cilastatin	≤0.25 (S)	≤0.25 (S)	≤0.25 (S)	≤0.25 (S)
Meropenem	NT	≤0.25 (S)	NT	NT
Amikacin	NT	≤2 (S)	NT	≤2 (S)
Gentamicin	≤1 (S)	≤1 (S)	≤1 (S)	≤1 (S)
Tigecycline	NT	NT	NT	NT
Tobramycin	≤1 (S)	≤1 (S)	≤1 (S)	≤1 (S)
Trimethoprim-sulfamethoxazole	≤2/38 (S)	≤2/38 (S)	≤2/38 (S)	≤2/38 (S)

^*a*^ Isolate numbers correspond to case numbers in Table 2. Values displayed are either minimum inhibitory concentrations (MIC, µg/mL) or zone diameters (mm). MIC results were generated by the Vitek2 automated system and zone diameters by disk diffusion. S, susceptible; I, intermediate; R, resistant; NT, not tested.

^*b*^ Index case, highlighted with gray shading.

^*c*^ Disk diffusion zone interpreted with CLSI M100 cefazolin breakpoints for systemic infections caused by *E. coli, K. pneumoniae,* and *P. mirabilis* for informational purposes only.

^*d*^ Added for informational purposes only. Nitrofurantoin results were suppressed in these isolates. Nitrofurantoin is only reported on urine isolates and indicated for the treatment of uncomplicated cystitis.

A nitrocefin disk (Hardy) was used to evaluate β-lactamase production in the *K. intermedia* isolate. Nitrocefin is a chromogenic cephalosporin used to rapidly detect β-lactamases that inactivate “penicillinase-labile penicillins” such as ampicillin. The β-lactamase negative, ampicillin-susceptible *E. coli* ATCC 25922 strain and the TEM-1 positive, ampicillin-resistant *E. coli* ATCC 35218 strain were used for quality control. The *K. intermedia* isolate tested negative for β-lactamase production and was susceptible to ampicillin (MIC <2 µg/mL). It was also catalase-positive and -negative for indole and oxidase production. The negative indole reaction reportedly differentiates *K. intermedia* from other *Kluyvera* species (14).

In a retrospective 10-year culture review, we identified three additional cases at our institution from 2023 to 2024 (Table 2), including two severe infections (isolated from sputum and urine) and one case of asymptomatic bacteriuria. The three strains were resistant to cefoxitin and ampicillin, in alignment with previous reports of intrinsic resistance to these agents but diverging from the pan-susceptible *K. intermedia* in our case report (Table 1). All four strains isolated at our institution tested susceptible to ceftriaxone, cefepime, piperacillin-tazobactam, carbapenems, trimethoprim-sulfamethoxazole, and fluoroquinolones.

**TABLE 2:** Cases of *K. intermedia* infections in a tertiary care center in Southern California from 2014 to 2024 (cases 1-4) and a case report of *K. intermedia* from published literature (case 5)^*a*^

Case	Year	Age/sex	Past medical history	Source	Sample	Polymicrobial culture	Treatment	Outcome
1	2022	74F	Neurogenic bladder and recurrent UTIs	Urinary tract infection	Urine	Yes	Trimethroprim-sulfamethoxazole	N/A
2	2023	72M	Chronic bronchiectasis	Community-acquired pneumonia	Sputum	Yes	Cefepime	Death on hospital day 10
3	2023	78F	Solitary kidney and nephrolithiasis	Nephrolithiasis	Urine	Yes	Imipenem-cilastatin	Underwent procedure without complications
4**^*b*^**	2024	61M	Uncontrolled diabetes	Acute cholecystitis	Blood	No	Piperacillin-tazobactam, levofloxacin	Discharge home
5**^*c*^**	2023	66F	Diabetes and pyelonephritis	Pyelonephritis	Blood	N/A	Piperacillin-tazobactam	Discharge home

^*a*^ UTIs, urinary tract infections; F, female; M, male; N/A, not applicable.

^*b*^ Index case, highlighted with gray shading.

^*c*^ External case reported by Inoue et al. (13).

### Analysis of β-lactamase genes in publicly available *Kluyvera* whole-genome sequences

To assess the prevalence of chromosomal *bla*_KLU_ genes across *Kluyvera* species, we analyzed whole-genome sequences on the National Center for Biotechnology Information (NCBI) database. The NCBI Pathogen Detection Isolate Browser Tool and the general NCBI Genome Database search function were used to identify a total of 162 unique, complete, high-quality *Kluyvera* genomes, including 58 *K*. *ascorbata* genomes and 41 *K*. *intermedia* genomes. The genomes belonged to diverse strains isolated from humans (infection and colonization), animals, plants, communities, and nosocomial environmental sources. AMRFinderPlus (v4.0.22) and PlasmidFinder (v2.1) were used to identify β-lactamase genes and predict their genomic location as chromosomal or plasmid-borne. Available publications associated with each genome project were reviewed for additional context. The results of our analysis are summarized in Table 3.

**TABLE 3:** Distribution of notable chromosomal or plasmid-mediated β-lactamase genes identified in publicly available *Kluyvera* genomes on the NCBI database across species

Species	Total genomes	Chromosomal *KLU* β-lactamase genes, % (*n*)	Plasmid β-lactamase genes, % (*n*)			
			ESBL (e.g. CTX-M, TEM, SHV, OXA-1, DHA)	KPC	OXA-48 like	NDM, IMP
*Kluyvera ascorbata*	58	100% (58)	32.8% (19)	32.8% (19)	10.3% (6)	5.2% (3)
*Kluyvera intermedia*	41	0%	73.2% (30)	63.4% (26)	0	0
*Kluyvera cryocrescens*	24	100% (24)	45.8% (11)	20.8% (5)	0	33.3% (8)
*Kluyvera georgiana*	12	33.3% (4)	75% (9)	8.3% (1)	0	0
*Kluyvera sichuanensis*	11	100% (11)	18.2% (2)	63.6% (7)	9.1% (1)	0
*Kluyvera* spp.	16	62.5% (10)	25% (4)	0	12.5% (2)	0
All *Kluyvera* species	162	66.1% (107)	46.3% (75)	35.8% (58)	5.6% (9)	6.8% (11)

Chromosomal *bla*_KLU_ genes were not uniformly present in all *Kluyvera* species. Of all *Kluyvera* genomes, 66.1% (107/162) harbored chromosomal *bla*_KLU_ genes. Strikingly, although chromosomal *bla*_KLU_ genes were absent in all 41 *K*. *intermedia* genomes, they were present in all 58 *Kluyvera ascorbata,* 24 *Kluyvera cryocrescens,* and 11 *Kluyvera sichuanensis* genomes (Table 3). Of the *Kluyvera georgiana* genomes, 33.3% (4/12) harbored chromosomal *bla*_KLU_ genes. Additionally, 62.5% (10/16) of the genomes from unclassified *Kluyvera* species harbored chromosomal *bla*_KLU_ genes. Diverse plasmid-borne β-lactamase genes were also identified. Plasmid-borne genes encoding for ESBLs, KPC, OXA-48, and metallo-β-lactamases were detected in 46.3%, 35.8%, 6%, and 6.8% of all *Kluyvera* genomes, respectively (Table 3). Plasmid *bla*_KPC_ and *bla*_ESBL_ genes were more prevalent in *K. intermedia* genomes relative to other opportunistic *Kluyvera* species which more often harbor genes encoding for OXA-48-like enzymes and metallo-β-lactamases (Table 3).

## DISCUSSION

Here, we report a case of an exceedingly rare opportunistic pathogen, *Kluyvera intermedia,* causing bacteremia and septic shock. *Kluyvera* are commensal gram-negative bacilli that colonize the gastrointestinal tract. The organism in this case likely translocated from the gastrointestinal or biliary tract into the bloodstream during the episode of cholecystitis, facilitated by the patient’s immunocompromised state due to uncontrolled diabetes. The patient was successfully treated with piperacillin-tazobactam and surgical source control. This case highlights the pathogenic potential of *K. intermedia,* which can cause invasive infections in immunocompromised patients.

The widespread use of MALDI-TOF in clinical laboratories has led to more rapid identification of rarer microorganisms that likely contributed to the increase in reports of *Kluyvera* infections. *K. ascorbata* (15, 16) are the most common species implicated in reports of adult and pediatric human infection, followed by *K. cryocrescens* and *K. georgiana* (1, 7). There is only one previous report to date describing *K. intermedia* infection in a 66-year-old woman with diabetes, hypertension, and cerebral hemorrhage who presented with bacteremia and septic shock secondary to obstructive pyelonephritis caused by a left ureteral calculus (13). Blood cultures grew *K. intermedia,* which was cephalosporin-resistant but susceptible to piperacillin-tazobactam. The patient improved with piperacillin-tazobactam, pressors, and ureteral stenting. The four cases from our institution and the previously published case report all involved patients above the age of 60 years with comorbid conditions that either made them immunocompromised or increased their risk of spontaneous infection and septicemia.

The *K. intermedia* isolate in our case report was unusually susceptible to all antimicrobials and lacked β-lactamase production, differentiating it from the previous case report (13). In current literature, *Kluyvera* are widely considered to be intrinsically resistant to ampicillin, first- and second-generation cephalosporins due to chromosomal *bla*_KLU_ genes (3, 9, 13, 16). Blood culture molecular identification panels in clinical laboratories have been reported to falsely detect *bla*_CTX-M-15_ in *Kluyvera* due to high gene sequence homology (84-100%) between *bla*_CTX-M_ and *bla_KLU_* genes despite their distinct hydrolysis profiles (6, 17). In our case, the ePlex blood culture molecular identification panel misidentified *K. intermedia* as “*Enterobacter* (non*-cloacae* complex),” likely due to the close phylogenetic relatedness of the organisms but did not detect *bla*_CTX-M_ 0 (3). Interestingly, the isolates from the three other cases at our institution were resistant to ampicillin and cefoxitin. These were identified by the Vitek MS MALDI-TOF with scores > 2.0. *K. ascorbata, K. intermedia,* and *K. cryocrescens* were added as newly claimed organisms to the Vitek MS database v3.2 in 2019, which may contribute to *Kluyvera* isolates being increasingly identified in clinical laboratories. However, genomic diversity and the rapidly evolving taxonomic landscape of *Kluyvera* likely complicate the reliability of a species-level identification.

*Kluyvera* are most well-known in literature for their chromosomal *bla*_KLU_ genes, which are reportedly ancestors of CTX-M enzymes (17). Plasmid-encoded CTX-M enzymes are traditionally thought to originate from the mobilization of chromosomal *bla*_KLU_ genes in *Kluyvera*. However, previous *in silico* analyses have also suggested that mobile *bla*_CTX-M_ genes may have circulated among various *Kluyvera* species and thus could have been incorporated into the organisms after the species diverged (17). Thus, the precise origin of CTX-M enzymes may still be unknown. This is supported by our analysis of publicly available *Kluyvera* genomes on the NCBI database showing that 0% of *K. intermedia* genomes harbor chromosomal *bla*_KLU_ genes that are present in all *K. ascorbata, K. cryocrescens,* and *K. sichuanensis* genomes (Table 3). A draft genome published in 2017 of a *K. intermedia* isolate from a patient with a pancreatic abscess also mentioned the lack of a chromosomal counterpart to CTX-M enzymes (18). The presence of chromosomal *bla*_KLU_ genes varies in genomes from other species like *K. georgiana* (33.3%) or those without a species-level classification yet (62.5%). The increased use of higher-quality, short- and long-read whole genome sequencing has added substantial complexity and granularity to *Kluyvera* nomenclature. Contemporary genomic studies have proposed taxonomic revisions to the *Kluyvera* genus, including the reassignment of *K. intermedia* lacking *bla*_KLU_ genes to the *Phytobacter* genus due to high phylogenetic relatedness (19).

Over the last decade, advancements in whole-genome sequencing and clinical microbiology diagnostics have improved our understanding of the *Kluyvera* genus. This case highlights species-specific genomic variations in the *Kluyvera* genus that impact antimicrobial susceptibility of pathogenic isolates. It questions the current scientific paradigm in which *Kluyvera* are considered to possess genus-wide intrinsic resistance to penicillins and early generation cephalosporins. This case also adds important clinical relevance to previous genomic findings suggesting that taxonomic updates to the *Kluyvera* genus might be warranted, which can also improve our understanding of the true origins of modern CTX-M enzymes. Clinical laboratories should ensure that their MALDI databases are up to date for improved accuracy in the detection of rare pathogens like *K. intermedia*. In summary, these findings urge clinicians to use phenotypic susceptibility testing results to guide treatment of *Kluyvera* infections to spare higher-level β-lactams which target organisms that produce penicillinases or true ESBLs.
